# Machine Learning to Analyze Single-Case Data: A Proof of Concept

**DOI:** 10.1007/s40614-020-00244-0

**Published:** 2020-01-21

**Authors:** Marc J. Lanovaz, Antonia R. Giannakakos, Océane Destras

**Affiliations:** 1 École de Psychoéducation, Université de Montréal, C.P. 6128, succursale Centre-Ville, Montreal, QC H3C 3J7 Canada; 2 Manhattanville College, Purchase, NY USA; 3 Polytechnique Montréal, Montreal, Quebec Canada

**Keywords:** AB design, Artificial intelligence, Error rate, Machine learning, Single-case design

## Abstract

Visual analysis is the most commonly used method for interpreting data from single-case designs, but levels of interrater agreement remain a concern. Although structured aids to visual analysis such as the dual-criteria (DC) method may increase interrater agreement, the accuracy of the analyses may still benefit from improvements. Thus, the purpose of our study was to (a) examine correspondence between visual analysis and models derived from different machine learning algorithms, and (b) compare the accuracy, Type I error rate and power of each of our models with those produced by the DC method. We trained our models on a previously published dataset and then conducted analyses on both nonsimulated and simulated graphs. All our models derived from machine learning algorithms matched the interpretation of the visual analysts more frequently than the DC method. Furthermore, the machine learning algorithms outperformed the DC method on accuracy, Type I error rate, and power. Our results support the somewhat unorthodox proposition that behavior analysts may use machine learning algorithms to supplement their visual analysis of single-case data, but more research is needed to examine the potential benefits and drawbacks of such an approach.

Visual analysis is the most commonly recommended method for analyzing single-case data (Dart & Radley, 2017; Lane & Gast, 2014). This approach entails making a series of decisions regarding changes in level, trend, variability, overlap, and immediacy between contrasting phases in a graph (Kratochwill et al., 2010). Differences in these dimensions of behavior across phases typically suggest a change in the dependent variable and provide support for the existence of a functional relation. Although a recent study found an interrater agreement of nearly 90% for a small dataset for which a context was provided (Ford, Rudolph, Pennington, & Byiers, 2019), researchers have typically found visual analysis to have inadequate interrater agreement (Fisher, Kelley, & Lomas, 2003; Matayas & Greenwood, 1990; Ninci, Vannest, Willson, & Zhang, 2015). In particular, interrater agreements when evaluating AB graphs typically vary from 60% to 75% (DeProspero & Cohen, 1979; Fisher et al., 2003; Ninci et al. 2015).

To address this issue of reliability, several researchers have developed structured aids to improve the accuracy of visual analysis. Three common aids that can be used with AB designs are the split-middle line for identifying trend (White & Haring, 1980), the dual-criteria method (DC) and the conservative dual-criteria method (CDC; Fisher et al., 2003). The split-middle method involves drawing a regression line (i.e., trend) through the baseline data. When making comparisons across phases, the baseline trend line is superimposed onto the subsequent treatment phase to determine if a change in trend has occurred. The split-middle method has the advantages of not requiring advanced calculations and is easy to implement, but it is limited insofar as it only supplements decisions regarding trend and does not consider the other behavioral dimensions needed in visual analysis (Manolov & Vannest, 2019).

In response to these limitations, Fisher et al. (2003) developed the DC and CDC methods. The DC method uses the split-middle method as its starting point but refines it by including a second level line that is determined by the baseline mean. These two lines are then overlaid on the treatment condition. If a specified number of data points fall above or below these lines in the desired direction of change, the visual analyst concludes that a change in the dependent variable has occurred. The CDC method is a more conservative version of the DC method that shifts the height of the two criterion lines by 0.25 standard deviations from the baseline data. The CDC method was created to compensate for the high rate of Type I errors made by the DC method when autocorrelation was present in the datasets.

In a demonstration of effectiveness, Fisher et al. (2003) had novice raters evaluate simulated graphs using the DC method. Interrater agreement increased from 71% to 95% following training on the DC method. Moreover, Lanovaz, Huxley, and Dufour (2017) reported that the DC method provided adequate control over Type I error rate with nonsimulated data. In a recent study, Wolfe, Seaman, Drasgow, and Sherlock (2018) evaluated the rate of agreement between the CDC method and expert visual analysts on 66 AB tiers from published multiple baseline graphs. Their findings show that mean agreement between the CDC method and expert visual analysts was 84%, indicating that the CDC method may still yield incorrect classifications in one of six cases. Another limitation is that the DC and CDC methods do not directly address issues related to the variability and immediacy of behavior changes.

Manolov and Vannest (2019) developed the visual aid implying an objective rule (VAIOR) method. The VAIOR provides objective operational rules to determine if contrasted phases in a graph demonstrate changes while considering, trend, variability, overlap, and immediacy. This new structured aid to visual analysis eliminates the need for subjective decision making, but agreement with expert visual analysts remains unknown. In addition, the procedure may not be as useful when baseline data contain too much variability to fit a meaningful trend line. A major drawback of VAIOR is that it produces high levels of Type I errors with datasets having fewer than 10 data points in each phase, which are common in single-case designs.

Given these limitations, behavior analysts still need tools that reduce the subjectivity of decision making while performing adequately with the smaller datasets that are common in single-case research and practice (Shadish & Sullivan, 2011). One potential solution may be to use machine learning. Machine learning, which falls within the broad domain of artificial intelligence, involves the use of algorithms to “learn” a model that will produce predictions based on data provided by the analyst. In the case of AB graphs, machine-learning algorithms may train models that predict whether the intervention introduced in Phase B produced a clear change in behavior or not, but no research has examined this possibility. Thus, the purpose of our study was to (1) examine correspondence between visual analysis and models derived from different machine-learning algorithms, and (2) compare the accuracy, Type I error rate and power of each of our models with those produced by the DC method.

## General Method

Our study tested four of the most popular machine-learning algorithms: stochastic gradient descent classifiers, support vector classifiers, random forest classifiers, and dense neural networks. Given that the intended readers for the current article are behavioral practitioners and researchers, we do not present the complex mathematical formulas underpinning these algorithms. Nevertheless, the following sections provide brief, simplified explanations on how machine-learning algorithms work. Readers who want to learn more about machine-learning algorithms may consult the references cited in each subsection.

### Machine-Learning Algorithms

To facilitate comprehension, Table 1 provides descriptions of some machine-learning terms used throughout the subsequent sections. To draw a parallel with behavior analysis, supervised machine learning is similar to multiple exemplar training. The analyst provides sets of features (exemplars) and their corresponding labels (correct responses), which are trained using an algorithm (training procedure). The algorithm trains a model (a learner) to predict the labels based on the features provided (like a learner who is trained to determine whether different images are exemplars of a cat). As in behavior analysis, the ultimate goal of machine learning is for this model to correctly label sets of features that were never used during training (generalization to novel, untrained exemplars).

**Table 1:** Some Machine-Learning Terms

Term	Description
Algorithm	At its broadest, an algorithm is a set of instructions that provides a solution to a problem. In the case of machine learning, these instructions are typically statistical computations that aim to predict the value of a label.
Hyperparameter	A hyperparameter is a value or a function of the algorithm that is set by the experimenter. That said, the experimenter may compare the results produced by different combinations of hyperparameters using a validation set in order to select the best model.
Features	The features represent the input data, which are transformed by the algorithm to provide a prediction. In the current study, the eight features were: (1) the mean of points in Phase A, (2) the mean of points in Phase B, (3) the standard deviation of points in Phase A, (4) the standard deviation of points in Phase B, (5) the intercept of LSRL for Phase A, (6) the slope of LSRL for Phase A, (7) the intercept of LSRL for Phase B, and (8) the slope of LSRL for Phase B.
Label	The label is what the algorithm is trying to predict from a set of features. The current study has a single binary label: clear change (1) or no clear change (0).
Model	A model refers to a specific algorithm with fixed hyperparameters and parameters.
Parameters	The parameters are the values that are fit to the training data (e.g., weights).
Test set	A set of features and labels that are never used in fitting the parameters or fixing the hyperparameters. This set is used to test for generalization.
Training set	A set of features and labels used during training to fit the best parameters to the model.
Validation set	A set of features and labels that are used to compare the accuracy of different combinations of hyperparameters. The validation set is not used during training to set the parameters. It simply allows the selection of the model that produces the best generalization.

Note: LSRL = Least squares regression line

In more technical terms, machine-learning algorithms produce models, which are comprised of hyperparameters and parameters specific to the algorithm. Hyperparameters are values or equations within the initial machine-learning algorithm that are typically set and manipulated by the experimenter prior to training. The machine-learning algorithm then uses these hyperparameters to fit parameters (e.g., weights) to the data that will best predict the labels of the dataset. The values of the parameters of the model determine its accuracy whereas the hyperparameters provide information as how the algorithm should process the data. Table 2 presents the values of the hyperparameters that we set and manipulated as part of the current study (see below for explanation of each hyperparameter). To train the model, the experimenter must also provide a dataset with features and labels. In our case, the features are descriptive statistics extracted from the data points in the AB graphs and the label is a binary variable (0: no clear change, 1: clear change). During testing, the model produces an output, which are predictions based on untrained data. These predictions have the same format as the labels (e.g., binary variable).

**Table 2:** Constant and Variable Hyperparameters for Each Machine-Learning Algorithm

Algorithm	Hyperparameters	
	Constant Values	Values Tested
SGD	Loss: Logistic regression Penalty: ElasticNet	Learning rate: 10^-5^–10^-2^ Epochs: 5–1,000 by 5
SVC	Kernel: Radial basis function	Penalty C term: 1, 10, 100 Gamma: 10^-5^–10^-1^
Random forest		Estimators: 10–190 by 10
DNN	Early stopping: No improvement in loss function for 30 epochs Learning rate optimizer: Adam Loss: Binary cross entropy Neuron activation function: ReLu Output activation function: Sigmoid	Neurons: 2^3^–2^6^ Hidden layers: 0, 1, 2, 4, 6

Note: SGD = stochastic gradient descent; SVC = support vector classifier; DNN = dense neural network

#### Stochastic gradient descent classifiers

Stochastic gradient descent in machine learning is analogous to shaping in behavior analysis. That is, the algorithm produces successive predictions (approximations) of the labels until these predictions best matches the true labels (terminal behavior). During training, stochastic gradient involves only keeping the model that produces the best accuracy while discarding the others (as differential reinforcement does with behavior). In mathematical terms, the algorithm 1) multiplies the input (features) by weights (parameters), 2) transforms the previous products using a function that produces predictions, and 3) updates the weights by applying a correction related to the gradient of the error on the predictions for the next iteration (Witten, Frank, Hall, & Pal, 2017). The algorithm runs in a loop and updates its parameters at the end of each iteration (i.e., one epoch) to minimize the error of the predictions. In our models, the function that produced the predictions was a logistic regression and we manipulated the magnitude of the correction applied to the gradient across each epoch (learning rate). If the stochastic gradient descent were to run indefinitely, the error would eventually reach zero (i.e., perfect prediction). On the other hand, running too many epochs may produce models that fail to generalize to novel (untrained) data (i.e., overfitting). To address this issue, we varied the number of epochs to select the model that produced the best generalization to the validation set and added a penalty hyperparameter, ElasticNet, which is designed to minimize overfitting.

#### Support vector classifiers

**Fig. 1:**
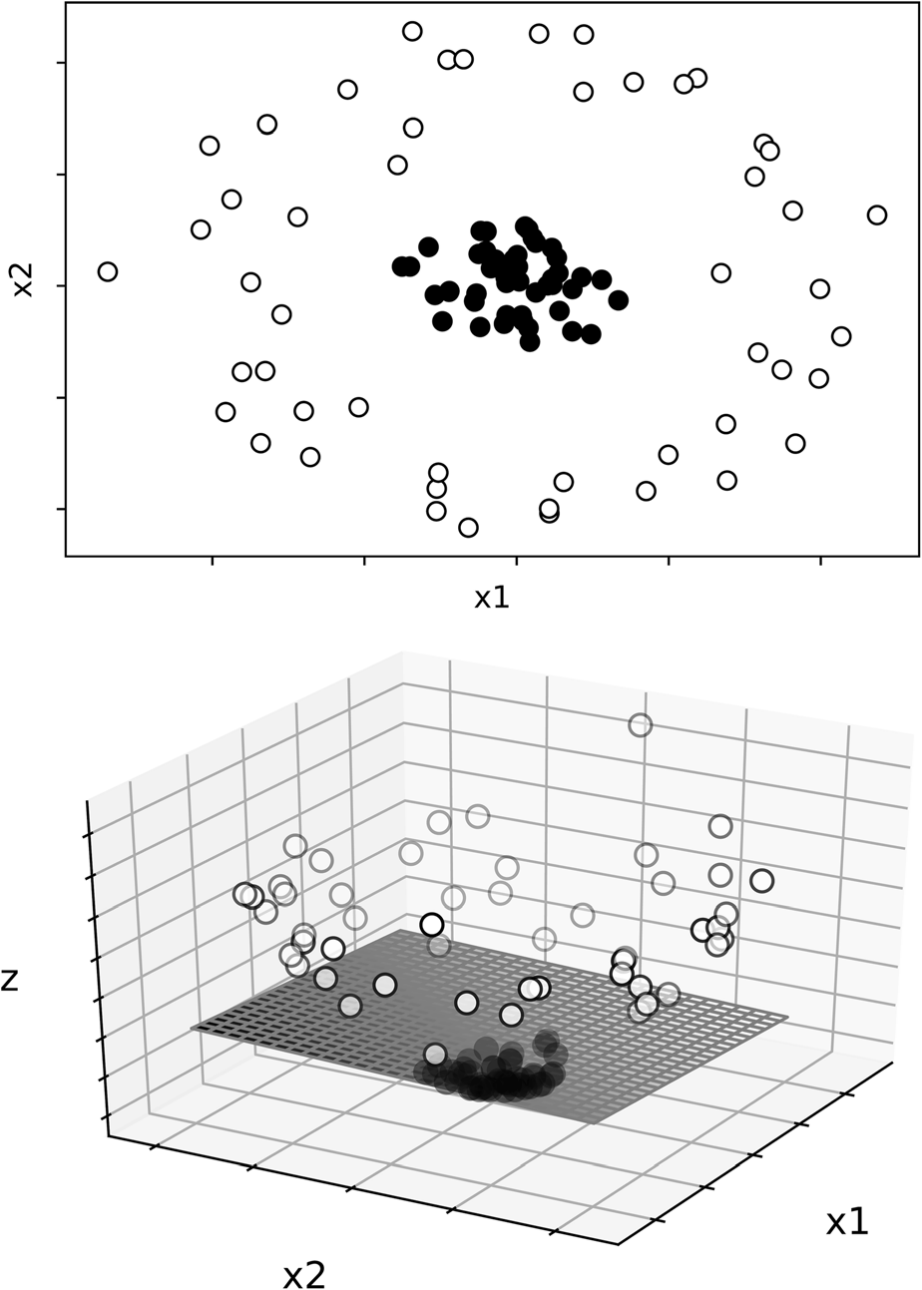
The upper panel shows a two-dimensional graph representing two features: x1 and x2. Closed points represent one category and opened points a different category. The lower panel depicts the addition of a higher dimension (z) and a linear plan that separates the two categories

A support vector classifier works by producing a hyperplane in a higher-dimensional space to split the dataset according to the categorization of the initial labels (Witten et al., 2017). Figure 1 shows a simple example of how a hyperplane may separate the data in two categories. The upper graph depicts the values of two categories (i.e., closed point and opened points) with two features (i.e., x1 and x2). It is clear that a simple linear equation (i.e., a straight line) cannot efficiently separate the two categories. The support vector classifier uses a function to separate the data in a higher dimension (see lower panel). Now, a plane can separate the data depicted into two categories: the closed points are below the plane whereas the opened points are above. When this separation is conducted in higher dimensions (as in the case when having multiple features), we refer to these planes as hyperplanes. Support vector classifiers attempt to maximize the distance between the hyperplane and the data in each category. One hyperparameter for support vector classifiers is the kernel, which is a set of functions that transforms the data to allow separation. The radial basis function kernel, which we used as part of the current study, also has two hyperparameters: the C penalty term and gamma. The C penalty term modifies the margin of the separation of the hyperplane whereas gamma influences the weight of a single training exemplar. Both hyperparameters are manipulated together to minimize overfitting.

#### Random forest classifiers

**Fig. 2:**
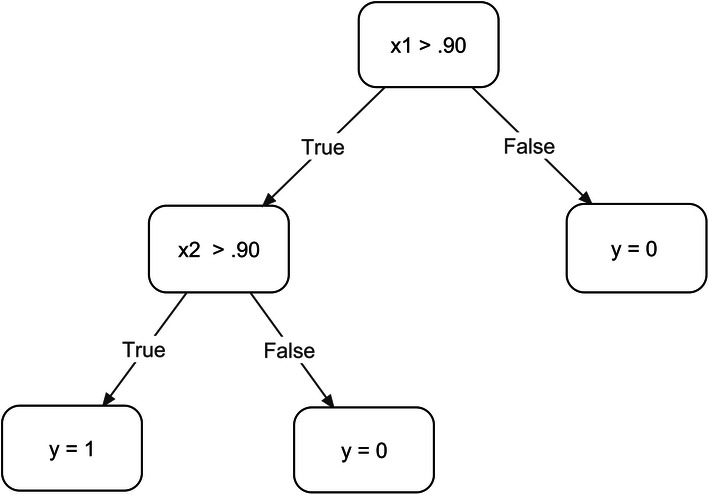
A decision tree where the percentage of correct responding in the next-to-last (x1) and last (x2) sessions are used to decide whether a concept is mastered (y = 1) or not mastered (y = 0)

Random forests are made up of a series of decision trees that are constructed during training (Breiman, 2001). As an example, assume that you are conducting discrete trial instruction with a child with autism and that you consider that the child has mastered a target when she responds correctly on more than 90% of trials for two consecutive sessions. The output (y) is whether the child has mastered the target (y = 1) or has not mastered the target (y = 0). The model has two features: x1 represents the percentage of correct responding of the child on the next-to-last session and x2 the same measure on the last session. Figure 2 depicts the process as a decision tree. Random forests are similar except that the trees are automatically produced by algorithms containing a randomizing component (and not by some conceptual logic). Random forests create decision trees by taking a random sample with replacement of examples from the training set, selecting randomly permutated features at each decision node, and choosing the feature that produces the most homogeneous groups following the split. Then, the algorithm selects a new group of examples to sample a new tree and the process repeats itself. The algorithm produces many independent trees, which are referred to as a forest. The final classification is either the average prediction of all trees in the forest or the prediction that occurs most often. When developing a random forest classifier model, the number of trees in the forest are a hyperparameter known as estimators.

#### Dense neural networks

**Fig. 3:**
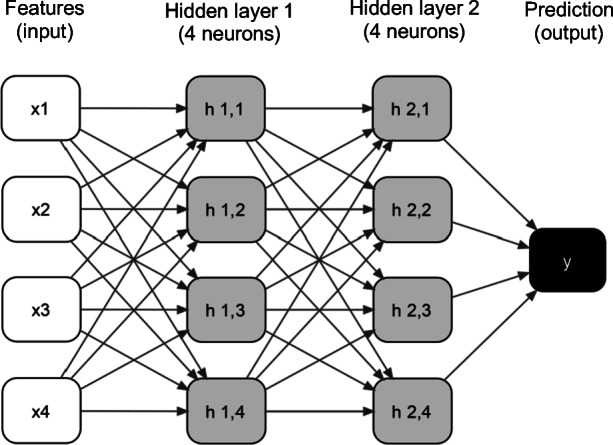
Dense neural network with four features, two hidden layers with four neurons each and a prediction

Dense neural networks are artificial neural networks in which neurons are arranged in fully connected layers. In the context of machine learning, a neuron is a decision-making unit that receives some input and transforms it into some output using an activation function (Goodfellow, Bengio & Courville, 2016). Figure 3 shows an example of a dense neural network. A dense neural network contains an input layer (i.e., the features), hidden layers with neurons, and an output layer (i.e., prediction). The hidden layers transform the data received from the input (or from other hidden layers) before outputting them to the next layer in the network. These hidden layers allow the algorithm to model complex relationships between features in the dataset. In a dense neural network, each neuron in a layer is connected to and receives input from every neuron in the layer preceding it. The activation function is a hyperparameter that produces a nonlinear transformation of the data between the layers and prior to producing the final output. During each epoch, the parameters of the model (i.e., weights) are updated by the loss function that computes the error and retropropagates it by an amount proportional to the learning rate (akin to a stochastic gradient descent with many layers of weights). Both the loss function and learning rate optimizer are hyperparameters. Our dense neural network models also included an early stopping hyperparameter, which instructs the network at which epoch it should stop training.

## Study 1: Correspondence with Visual Analysis

As behavioral researchers and practitioners may be unfamiliar with machine-learning procedures, we provide a step-by-step tutorial to replicate our study on the OpenScience Framework (OSF).^1^ The tutorial also includes explanations on how to analyze single or multiple graphs using the best models from this study. Our code, datasets, and best models are also available in the same repository.

### Datasets

#### Complete nonsimulated dataset

For our initial study, we limited our analyses to graphs with three points in phase A and five points in phase B because (1) graphs with fewer points are typically more difficult to analyze visually and statistically (Manolov & Vannest, 2019) and (2) this arrangement represents the minimum number of points under which the DC method performs satisfactorily (Lanovaz et al., 2017). Our graphs all came from a dataset previously described by Lanovaz, Turgeon, Cardinal, and Wheatley (2019). The Lanovaz et al. (2019) dataset contained a total of 501 ABAB graphs extracted from theses and dissertations.

To obtain AB graphs from the original dataset, we coded R to first split each ABAB graph into three AB graphs (i.e., first A phase with first B phase, first B phase with second A phase, second A phase with second B phase) and recorded the expected direction of change (i.e., increase or decrease) for each graph. Albeit theoretically a BA graph, we also refer to the middle graph (i.e., first B phase with second A phase) as an AB graph; however, we reversed the expected direction of change when compared to the two other AB graphs extracted from the same ABAB design. The program rejected AB graphs that contained fewer than five points in the second phase. When graphs contained more data points than the required number (i.e., three in Phase A and five in Phase B), we used only the last three points of Phase A and the first five points of Phase B so that the points on either side of the phase change lines remained consistent. In total, our extraction yielded 1,070 AB graphs for training, validation, and testing.

#### Nonsimulated dataset with agreements only

As described below, two observers independently categorized the graphs as either showing a clear change or no clear change. In the dataset with agreements only, we only kept graphs for which the two raters agreed on the labels to examine whether a consensus-based approach would train more accurate models. Because the interrater agreement was 91%, this dataset contained 970 graphs.

### Procedures

#### Labeling the nonsimulated dataset

**Fig. 4:**
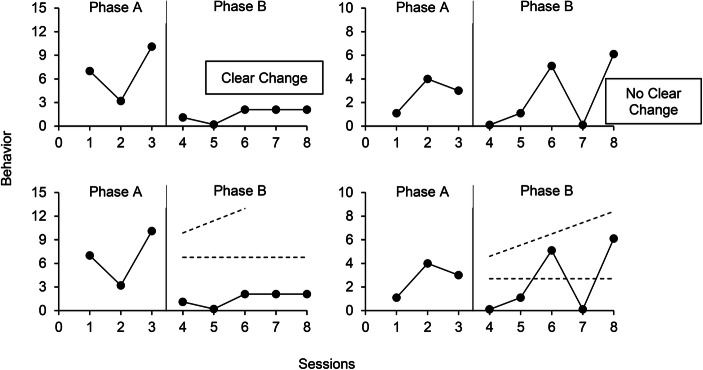
Graphs showing a clear change and no clear change (upper panels) and applications of the dual-criteria method (bottom panels)

The first author, a professor and doctoral-level certified behavior analyst who conducts single-case research, independently visually analyzed each of the 1,070 nonsimulated AB graphs by conducting a qualitative analysis of trend, immediacy of change, level change, and variability (Kratochwill et al., 2010). This analysis was conducted prior to applying the machine-learning algorithms. For each graph, he responded to the following question: “Would the change observed from Phase A to Phase B be indicative of functional control of the behavior in the planned direction (i.e., increase or decrease) if it were reversed and replicated?” A positive response led to the graph being categorized as displaying a clear change. Otherwise, the AB graph was categorized as showing no clear change. Figure 4 (upper panels) shows an example of a graph showing a clear change and another graph showing no clear change. Overall, the first author categorized 49% of graphs as showing no clear change and 51% as showing a clear change. The second author, also a professor and doctoral-level certified behavior analyst who conducts single-case research, independently checked interrater agreement by categorizing all 1,070 graphs using the same procedures. As indicated earlier, the agreement between the two raters was 91%.

#### Preparing the data

**Fig. 5:**
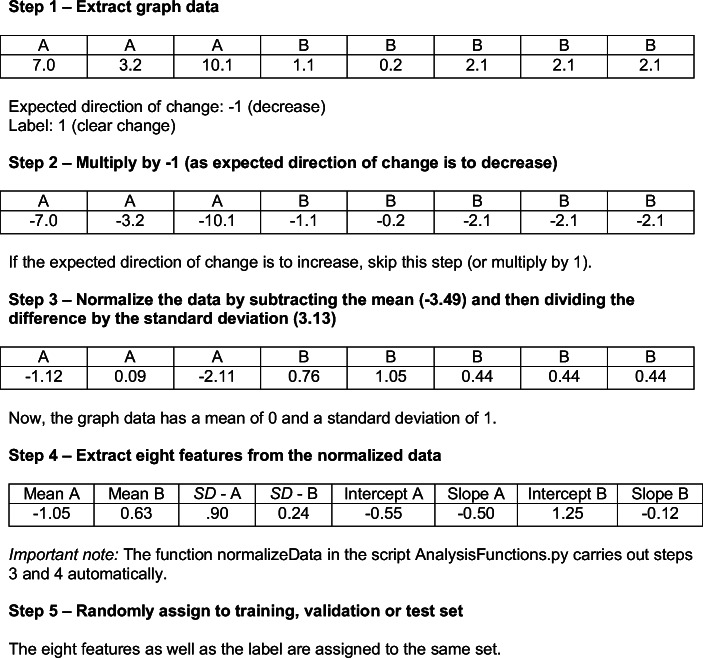
Normalization of graph data and extraction of features prior to training, validation, and testing

Prior to training, validating, and testing the models, we normalized the data and extracted features as each graph used different types of measures with ranges that varied widely. Figure 5 shows a sequence of the steps involved in normalizing the data and extracting the features from each graph. The data in the example are from the graph depicted in the left panel of Fig. 4. First, we extracted the data from the graph, which had already been done in a previous study (see Lanovaz et al., 2019). If the purpose of the intervention depicted on the graph was to reduce the target, the program multiplied all values by -1 so that all trends and patterns were reversed. Next, our program normalized the data so that each graph now had a mean of 0 and a standardized deviation of 1. Finally, our code produced a feature vector for the data. The eight features extracted were: 1) the mean of points in Phase A, 2) the mean of points in Phase B, 3) the standard deviation of points in Phase A, 4) the standard deviation of points in Phase B, 5) the intercept of the least squares regression line (LSRL) for Phase A, 6) the slope of LSRL for Phase A, 7) the intercept of LSRL for Phase B, and 8) the slope of LSRL for Phase B. We used the previous eight features, rather than the actual values of the data points, because it allowed us to use our models with phases of varying lengths (see Study 2).

#### Training the models

To train the models and conduct the analyses, the first and third authors wrote programs in Python, which made use of the Scikit-Learn and Tensorflow machine-learning packages. Our program randomly divided each nonsimulated dataset into three subsets: the training set, the validation set, and the test set. The training sets contained 50% of the nonsimulated data (535 graphs for the complete dataset and 485 graphs for the dataset with agreements only), which were used to adjust the parameters of the model during training. We provided these data to each algorithm along with the labels established by the first author (i.e., clear change vs. no clear change) or the labels on which both raters agreed. The validation sets involved 25% of the datasets (267 graphs for the complete dataset and 242 for the dataset containing agreements only). Each algorithm computed accuracy on the validation sets for different combinations of hyperparameters (see Table 2 for values tested). Then, our program only kept the model with the hyperparameters that produced the highest accuracy on the validation sets. The procedure with the validation sets was designed to select models that did not overfit the training data. Finally, the test sets contained 25% of the original nonsimulated datasets (268 graphs for the complete dataset and 243 graphs on the dataset containing agreements only). We never used the test sets during training or validation. In behavior analytic terms, the test sets checked for generalization.

#### Testing the models

Following training, we only kept the models with the combinations of hyperparameters and parameters that produced the best accuracy on the validation tests. Then, we applied these models to the test sets to examine whether they could accurately identify changes in behavior on data never seen by the algorithms. Our analyses examined whether the results produced by each model corresponded with visual analysis.

### Analysis

In the first study, we measured correspondence between visual analysis and different models trained using (1) the complete dataset and (2) the dataset containing agreements only. In particular, our Python code computed the proportion of time the prediction of each model matched the result of the visual analysis for each dataset by adding the number of agreements between the visual analyst and the model, and dividing this sum by the total number of graphs. Our program computed three values: one for all test graphs, one for test graphs showing a clear change only, and one for test graphs showing no clear change only (according to the visual analysts).

To compare the values to a well-established aid to visual analysis, we also applied the DC method algorithm to each graph. We chose the DC method over the CDC as the former tends to produce higher power. As described earlier, the DC method involves tracing a continuation of the mean and trend lines from Phase A into Phase B (see bottom panels of Fig. 4). Because the purpose of the intervention on Fig. 4 was to reduce the behavior, we counted the number of points that fall below *both* lines. In this case, all points fall below both lines for the graph in the left panel and three points fall below both lines in the right panel. The cut-off value based on the binomial distribution is five. Therefore, only the left graph shows a clear change according to the DC method. In contrast with all other models, the DC method was applied to the original data (see Step 1 of Fig. 5) as it did not require the normalization of the data.

## Results and Discussion

Table 3 presents the hyperparameters of the best models, which were used to make the predictions. Table 4 shows the correspondence between visual analysis and the different models for the two datasets. For the complete dataset, overall correspondence with visual analysis for the DC method was approximately 87% whereas all models derived from machine learning produced overall correspondence above 90%. By far, the DC method had the lowest correspondence with visual analysis for datasets showing a clear change in the complete dataset. The stochastic gradient descent and the support vector classifiers produced higher correspondence with graphs showing a clear change whereas the random forest and dense neural networks produced better correspondence for graphs showing no clear change. A further analysis indicated that the majority of graphs on which visual analysis and machine learning disagreed were also graphs on which visual analysis and the DC method disagreed (i.e., 78% for stochastic gradient descent, 74% for the support vector classifiers, 85% for random forest, and 79% for dense neural networks). That said, machine-learning algorithms produced considerably fewer disagreements overall.

**Table 3:** Hyperparameters of Best Model for Each Dataset

Algorithm	Hyperparameter	Complete nonsimulated dataset	Nonsimulated dataset with agreements only
SGD	Learning rate	.0001	.00001
	Epochs	60	215
SVC	Penalty Term	10	100
	Gamma	.1	.01
Random Forest	Estimators	30	180
DNN	Neurons	16	16
	Hidden Layers	6	2

Note: SGD = stochastic gradient descent; SVC = support vector classifier; DNN = dense neural network

**Table 4:** Correspondence between Visual Analysis and Models Derived from Different Machine Learning for Test Sets Showing Clear Change and No Clear Change

Algorithm	Models trained with complete nonsimulated dataset			Models trained with nonsimulated dataset with agreements only		
	Overall	Clear change	No clear change	Overall	Clear change	No clear change
DC method	.869	.822	.914	.942	.948	.937
SGD	.914	.930	.899	.951	.948	.953
SVC	.929	.938	.921	.959	.948	.969
Random forest	.902	.868	.935	.947	.948	.945
DNN	.925	.899	.950	.963	.957	.969

Note: DC = dual-criteria; SGD = stochastic gradient descent; SVC = support vector classifier; DNN = dense neural network

For models trained with the dataset containing agreements only, all algorithms produced higher correspondence with visual analysis than the models trained with the complete dataset. The DC method still produced the lowest correspondence with visual analysis, but the difference was not as large as the one observed for the complete dataset. Although the results seem to suggest that the dataset with agreements only produced better models, the differential characteristics of the graphs in each dataset prevent us from reaching this conclusion. Because the agreement-only dataset excluded all disagreements between the two raters, graphs with ambiguous patterns were most likely left out of the test set. As such, we tested the agreement-only models on graphs that showed clearer, more stable patterns than those in the complete dataset (i.e., the graphs in the agreement only dataset may have been easier to analyze). Therefore, we kept the models derived from both datasets for our subsequent analyses to conduct additional comparisons.

## Study 2: Accuracy, Type I Error Rate, and Power of Models

One issue with examining control with nonsimulated data is that the labels need to be established by an expert human rater. However, one can never be certain that the effects detected on the nonsimulated data represent true effects. Therefore, we conducted further analyses with simulated datasets, which were never used during training.

### Datasets

#### Simulated dataset with a constant number of points

We used R code to generate 20,000 AB graphs with three points in Phase A and five points in Phase B. The R code generated an eight-point data series for each graph using the equation:

$$ {x}_t=a{x}_{t-1}+{\varepsilon}_t+c $$

where *x* was the univariate times series, *t* an index of time (i.e., one to eight), *a* the autocorrelation value, ε a normally distributed error term with a mean of 0 and a standard deviation of 1, and *c* a constant of 10 to avoid graphs with negative values. The autocorrelation value varied across graphs according to a normal distribution with a mean of .20, a standard deviation of .15, and a maximum of .80, which are based on the values reported by Shadish and Sullivan (2011) for single-case research. The program produced graphs in pairs that shared an autocorrelation value. For one of the graphs in each pair, our program added a standardized mean difference (SMD) to the last five points (i.e., points from Phase B) to simulate a treatment effect. The SMD is a measure of the magnitude of mean change from baseline to treatment (see Pustejovsky, 2018). Like the autocorrelation term, the SMD varied across graphs using a normal distribution with a mean of 6.27, a standard deviation 2.37, and minimum of 1.00. We based the SMD mean and standard deviation on the median and the fifth percentile rank observed in the nonsimulated dataset. We used nonparametric estimators because the distribution of SMDs was highly skewed. In the end, our simulated dataset included 10,000 graphs simulating no effect (true label = 0) and 10,000 graphs simulating a treatment effect (true label = 1). In other words, the graphs with no SMD added were considered as showing no clear change whereas those to which we added SMD were categorized as showing a clear change.

#### Simulated dataset with a variable number of points

To examine the generality of our procedures, we programmed R to generate 20,000 additional graphs. The procedures remained the same as those described for the simulated dataset with a constant number of points. The only exception was that the number of points per phase varied randomly (based on a uniform distribution) between 3 and 6 for Phase A, and between 5 to 10 for Phase B.

### Analysis

To examine generalization to simulated datasets, we used the best models as trained and validated using the nonsimulated data (i.e., the same models as Study 1) to make our predictions. In particular, our analyses monitored accuracy, Type I error rate, and power. Accuracy represents to what extent the predictions match the true labels of the datasets (i.e., clear change vs. no clear change). Computing accuracy involved adding the number of agreements between the true labels and the predictions of the models (i.e., clear change or no clear change) and dividing the sum by 20,000. Type I error rate represents false positives. In the analysis of single-case design, a false positive occurs when a behavior analyst erroneously concludes that a graph shows a clear change when no real change occurred (i.e., the variations are the result of chance). Computing Type I error rate consisted of dividing the number of disagreements between the true labels and the predictions of the models on the graphs showing no clear change (according to the true labels) by 10,000. Power represents the extent to which a prediction identifies an effect that is present. In our case, power is the proportion of time the model detects a clear change when a real change did occur. To compute power, our program divided the number of agreements between the true labels and the predictions on the graphs showing a clear change (according to the true labels) by 10,000.

## Results and Discussion

Table 5 presents the results for the simulated dataset with a constant number of points. Regardless of the models used, accuracy and power were consistently higher for the models derived from machine-learning algorithms than for the DC method. Type I error rates remained at 5% or 6% for all methods. The models trained with the complete dataset and those trained with agreements only produced similar outcomes. Table 6 presents the accuracy, Type I error rate and power on simulated data with variable number of points. All models derived from machine-learning algorithms produced better accuracy, Type I error rates, and power than the DC method. The DC method produced a power of 90% and a Type I error rate of 7% whereas all models produced power ranging from 92% to 95% and Type I errors rates lower than 3%. The models trained with the complete dataset consistently produced marginally better accuracy than the ones trained with the agreements only. Thus, the differential results observed across the two datasets in Study 1 appears to have been an artifact of the procedures. When models trained with different datasets were tested on similar simulated data, the differential outcomes in favor of the dataset containing agreements only disappeared.

**Table 5:** Accuracy, Type I Error Rate and Power of the DC Method and Models Derived from Different Algorithms on Simulated Data with Three Points in Phase A and Five Points in Phase B

Algorithm	Models trained with complete nonsimulated dataset			Models trained with nonsimulated dataset with agreements only		
	Accuracy	Type I error	Power	Accuracy	Type I error	Power
DC method	.909	.059	.878	.909	.059	.878
SGD	.948	.064	.959	.949	.050	.972
SVC	.943	.063	.949	.944	.054	.942
Random forest	.940	.054	.933	.937	.059	.932
DNN	.946	.051	.943	.935	.067	.938

Note: DC = dual-criteria; SGD = stochastic gradient descent; SVC = support vector classifier; DNN = dense neural network

**Table 6:** Accuracy, Type I Error Rate and Power of the DC Method and Models Derived from Different Algorithms on Simulated Data with Varying Number of Points Per Phase

Algorithm	Models trained with complete nonsimulated dataset			Models trained with nonsimulated dataset with agreements only		
	Accuracy	Type I error	Power	Accuracy	Type I error	Power
DC method	.913	.074	.901	.913	.074	.901
SGD	.963	.025	.952	.962	.018	.942
SVC	.961	.024	.947	.960	.020	.940
Random forest	.953	.022	.928	.947	.028	.923
DNN	.960	.017	.937	.954	.027	.935

Note; DC = dual-criteria; SGD = stochastic gradient descent; SVC = support vector classifier; DNN = dense neural network

## General Discussion

Overall, our proof of concept indicates that different models developed using machine-learning algorithms may produce higher accuracy than the commonly used DC method. On the nonsimulated datasets, the models derived from machine-learning algorithms consistently showed better correspondence with visual analysis than the DC method. All our models yielded higher accuracy and power than the DC method on nonsimulated datasets. Moreover, the models produced their best outcomes on all measures when analyzing graphs with varying phase lengths. Each machine-learning algorithm performed differently across datasets with no one consistently outperforming the others.

To our knowledge, our study is the first to examine the validity of using machine learning to analyze single-case data. The analyses clearly showed that the learned models produced generally acceptable control over potential errors in interpretation. It is interesting that our models produced the best accuracy and lowest Type I error rate on the simulated dataset with phases of variable lengths. This observation is consistent with other studies that have shown that graphs with more points per phase produce more accurate interpretations (e.g., Lanovaz et al., 2017; Levin, Ferron, & Kratochwill, 2012; Manolov & Vannest, 2019). From a practical standpoint, these findings support the generalizability of our results to AB designs containing three or more points in Phase A and five or more points in Phase B. Our results also extend prior research on the use of the DC method for the analysis of single-case data. Agreement between the expert rater and the DC method was .869, which is a value consistent with recent research on the topic (Wolfe et al., 2018).

As more research is conducted, practitioners and researchers may eventually use machine learning to supplement visual analysis. As observed in the current study, machine-learning models may produce more accurate interpretations of data than the DC method. If the labels were established by a group of experts using consensus, the models could potentially be used as tools to train future practitioners and researchers on analyzing single-case data. Another potential use for our machine-learning models is as decision-making tools in mobile health apps designed to teach or reduce behavior (Knight, McKissick, & Saunders, 2013; Zapata, Fernández-Alemán, Idri, & Toval, 2015). For example, an app may prompt a user to collect data prior and during the implementation of an intervention and then automatically detect whether the technological intervention was effective or ineffective, which could lead to personalized recommendations.

Despite the promising nature of our results, our study has some limitations that should be noted. First, we restricted our algorithms to basic machine-learning models and manipulated only a limited number of hyperparameters as the purpose of our study was to examine whether machine learning could produce higher accuracy than a well-established structured aid to visual analysis. Future research should examine how accuracy can be further improved by manipulating other hyperparameters or using more advanced neural networks (e.g., recurrent neural networks, convolutional networks; Chung, Gulcehre, Cho, & Bengio, 2014; Krizhevsky, Sutskever, & Hinton, 2012). Second, we did not examine the differential effects of autocorrelation and specific phase lengths on accuracy (e.g., Fisher et al., 2003; Lanovaz et al., 2017). As a next step, researchers should conduct parametric analyses of autocorrelation and phase length while also comparing the results with other structured or statistical aids for the analysis of AB or multiple baseline designs (Ferron, Joo, & Levin, 2017; Giannakakos & Lanovaz, 2019; Krueger, Rapp, Ott, Lood, & Novotny, 2013; Manolov & Vannest, 2019). A final limitation is the large amount of data, or exemplars, required to train machine-learning models. Our results indicate that the size of our nonsimulated dataset was sufficient to outperform the DC method. Nonetheless, more data would be needed to further increase power and reduce Type I error rate. In the end, machine-learning algorithms may be innovative tools to support behavior analysts in both research and practice, but researchers need to conduct further studies to better identify the potential benefits and drawbacks of such an approach.

## Footnotes

1 The link to the repository is: https://osf.io/6wcty/. To access the tutorial, simply click on tutorial.ipynb.
